# Magnetron Sputtering vs. Electrodeposition for Hard Chrome Coatings: A Comparison of Environmental and Economic Performances

**DOI:** 10.3390/ma14143823

**Published:** 2021-07-08

**Authors:** Antoine Merlo, Grégoire Léonard

**Affiliations:** Department of Chemical Engineering, University of Liège, Quartier Agora B6a Sart-Tilman, 4000 Liège, Belgium; g.leonard@uliege.be

**Keywords:** magnetron sputtering, electrodeposition, chromium coating, technology comparison, life cycle assessment, economic analysis

## Abstract

The coating of materials with specific films is widely used to improve material properties and many technologies exist to perform it. In the last few years, the replacement of wet electrodeposition processes has been continuously encouraged in the EU due to the problematic waste management linked to those processes. In this paper, magnetron sputtering is studied as an alternative to conventional electrodeposition by comparing the technologies’ environmental impacts and costs. From the study, it appears that while magnetron sputtering greatly reduces hexavalent chromium emissions over the production, it has an increased electricity consumption mostly due to its lower production capacity, thus leading to more greenhouse gas emissions. Furthermore, a short discussion on the quantification of the impact of hexavalent chromium emissions is conducted. Regarding costs, the electrodeposition process has a lower cost of investment and of consumables, but requires more work time for the different steps of the process, making the total price per functional unit roughly equal. However, the cost per functional unit strongly depends on assumptions on the required work time, for which a sensitivity study is performed. Finally, the impacts of these two competing coating processes are discussed to complete the technological comparison for the case of hard chromium deposition.

## 1. Introduction

With the current focus of the scientific world on sustainability, finding more environment-friendly, cost-effective alternatives to current processes and technologies has become one the most important goals of researchers all around the world. In particular, thin films and coatings are increasingly prominent in everyday life, thanks to the ever-growing interest in micro-electronics, screens, solar panels; for instance, the global number of smartphone users increased by 44% in just 4 years, from 2.5 billion in 2016 to 3.6 billion in 2020 [1]. In addition, while its share is still relatively low in the global mix, energy generation from solar panels which use thin films technology, is one of the fastest growing markets [2]. Many other applications, ranging from antimicrobial [3,4] to anti-corrosion coatings use thin films [5]. In the USA, the market size of the total metal plating industry is of 22.9 billion USD [6], and market size is similar in Europe [7].

In this paper, hard chrome coatings are studied. These coatings are, among other applications, widely used in the aerospace industry to improve the mechanical properties of many aircraft components: landing gears, engine bearings; these coatings increase the lifetime of the components by reducing friction and improving hardness, as well as corrosion and wear resistance. They also play a large role in the automotive industry for the same reasons. In addition, the use of chromium for decorative applications account for about 70% of the applications in the automotive coatings market [7]. Chromium plating makes up 40% of the automotive electroplating market for a total global revenue of 1.43 billion USD in 2015, with an expected compound annual growth rate of about 6% [8].

The main deposition process used is electrodeposition with hexavalent chromium. There is, however, a push from governmental institutions to forbid the use of the carcinogenic chromium trioxide (CrO_3_) used in the process. As such, viable alternatives to the electrodeposition process are highly sought after. In this context, it is relevant to study processes with little to no waste generation as alternatives to the electrodeposition process such as magnetron sputtering. However, magnetron sputtering is not the only alternative, as, for the deposition of chromium, one may think to the use of trivalent chromium, thermal evaporation or electron beam evaporation. Many more processes using a different coating material such as high-velocity oxyfuel (HVOF) coatings for instance could also be considered substitutes. While all these technologies deserve full attention, they are beyond the scope of this paper, which focuses on magnetron sputtering.

It is important, for decision makers to be able to rely on sound comparisons of technologies, that do not only focus on a single aspect (environmental impact, economic relevance…), but that they consider several of them in order to draw a picture as complete as possible. The present paper can thus be seen as a case study for technological comparison, combining life cycle assessment and economic considerations.

In this paper, a brief description of magnetron sputtering and electrodeposition is first made to highlight the context of the present work. Then, the assumptions used for describing the coating processes, along with description of the life cycle assessment (LCA) and techno-economic assessment (TEA) methodologies are detailed in the section dedicated to methods. The results of the LCA and TEA are then presented and discussed, followed by a discussion on the toxicity and potential human impacts. Finally, the paper concludes by highlighting the main challenges of magnetron sputtering in view of these results, as well as proposes some perspectives to improve this comparison of competing technologies.

## 2. State of the Art

### 2.1. Electrodeposition

Electrodeposition is a deposition technique that has been mentioned already in the 18th century [9], and for the case of hard chromium coatings, it is still the most used. This technique consists in immersing the substrate to be coated in a liquid electrolytic bath, and in applying a current to reduce metallic ions from the liquid onto the substrate’s surface, leading to film growth. The substrate must typically be metallic in order to conduct current. Steel is often coated using this process to improve hardness, wear and corrosion resistance as well as sliding. Vickers hardness of around 1000 MPa are usually attained using this process [10].

In Europe, the electrodeposition process has been declining in use. Chromium compounds are not manufactured in Europe anymore and are imported less than they were previously. For example, imports of chromite decreased by 18% between 2012 and 2016 [11]. In addition, chromium use has been banned in its hexavalent form for certain applications, due to its carcinogenic properties. More regulations are being implemented for the highly toxic hexavalent chromium, in particular to limit workers’ exposure to hexavalent chromium [12,13]. Those regulations could lead to the closing of several plating businesses.

Electrodeposition is a mature process, and recent research around the chromium plating technology has mostly targeted pulsed plating (PP), trivalent chromium deposition and waste water management. Each of those areas of research aims to overcome part of chromium plating’s drawbacks. In the case of PP, research seems to indicate that using pulsed current can improve the coating’s properties, such as adhesion, wear and corrosion resistance, current efficiency, microstructure and more [14]. That technology could also improve the current distribution during the process, leading to more homogeneous coatings. PP is often applied to potential alternatives to hexavalent chromium such as nickel, tin or trivalent chromium [15]. Trivalent chromium especially has gained traction in the latest years. That technology uses a less hazardous form of chromium and is already in commercial use for decorative coatings. However, thicker coatings are harder to attain using trivalent chromium and bath maintenance is more demanding than for hexavalent chromium baths [16]. The technology using Cr^III^, however, shows promise in the form of better current efficiency and deposition rate [17]. In recent developments, number of other alternatives to chromium plating, such as high-velocity oxyfuel (HVOF), or chemical or physical vapour deposition (CVD or PVD), are often investigated for a potential replacement [18,19]. Among these alternatives, magnetron sputtering is further studied in this article as a PVD technique.

### 2.2. Magnetron Sputtering

Sputtering technologies have been studied since the 1800’s [20,21]. The main mechanism for sputtering is the use of the material to be sputtered as a cathode target in a vacuum chamber with the chamber as the anode. By applying a current to this target, the gas in the chamber is ionized (creating a plasma) and the ions are accelerated towards the target. The bombardment of the target by the ions leads to the sputtering of the target and consequently, a flow of material is transferred from the target to the substrate in the chamber and film growth occurs. Ever since Albert Hull’s work [22], magnets have been used to form electronic traps whose goal is to increase the ionization rate near the target, and subsequently lead to the increase of sputtering rates. In addition, this gives the possibility to work at lower vacuum pressure, increasing the film quality thanks to the higher energies of the species [23]. The use of a magnetic field remains, today, the most widespread process for this technology.

Magnetron sputtering has been used for decades to produce chromium coatings [24], with hardness and mechanical properties comparable to electrodeposited chromium, or even higher [25]. However, it has not reached full commercial maturity yet. Most recent research in the field of magnetron sputtering addresses concerns on the industrialization of the technology. Indeed, while the sputtering technique allows a good flexibility and process control, the main drawbacks are its low deposition rate and energy efficiency. To overcome magnetron sputtering’s shortcomings, several research focuses have been identified. First, to increase deposition rates, work has been done to improve the ionization, for example by using Radio-Frequency (RF) electricity sources [26]. Other techniques have been used as well, such as the use of segmented targets [27], heated targets/substrates [28], or the introduction of a voltage bias on the substrate [29]. Furthermore, the technology is being developed with automation as a focal point, with digital control of the process and software development as a goal [30,31]. Efforts are also being made to model the process itself using Computational Fluid Dynamics [32], or Monte Carlo simulations [33]. Finally, another point of interest in the research concerning sputtering is the development of high-performance coatings, for which High-Powered Impulse Magnetron Sputtering (HiPIMS) is a promising technique. This technique consists in using a power supply generating very short (<100 µs) pulses with a high-power intensity (a few kW/cm^2^ compared to around 10 W/cm^2^ for typical DC magnetron sputtering). This increases the ionization rate and allows for a better control of ion bombardment and tailoring of the phases of the growing film [34].

### 2.3. Environmental and Techno-Economical Evaluation of Chromium Deposition

In the literature, LCA has been used to assess the environmental impact of electrodeposition and to compare it with potential coating alternatives such as HVOF [35], or combined plasma and laser treatment [36]. The impact of different treatments for the wastewater resulting from electro-deposition plating was also studied, both from the environmental and cost point of view [37]. However, while those studies are quite insightful on the electrodeposition process, they do not directly relate to sputtered chromium.

Benveniste et al. did an LCA study to compare different alternatives for surface functionalization [38], including chromium electrodeposition, SiOx plasma and ceramics PVD. However, as the nature and the thickness of the PVD coating is different to the electrodeposited chromium, the study is not relevant for comparing PVD and electrodeposition technologies both applied to chromium films as it takes an arbitrary coating functionality. Additionally, the study did not include a cost assessment. While it is widely accepted that magnetron sputtering has a lower deposition rate and is costlier than electrodeposition, that fact has rarely been thoroughly studied alongside an environmental assessment. Through fundamental comparison of technologies, the present study thus aims to explore this gap in the research.

## 3. Methodology

In the present chapter, the methodologies used to assess the environmental impacts and costs will be presented. They require the identification of a complete and justified inventory of inputs and emissions, as well as the definition of a functional unit for the comparison of technologies.

The LCA being a standardized methodology, the inventory of mass and energy flows as well as the definition of a functional unit are precisely described in international standards (see below). In the present work, the functional unit and process inventories will be kept unchanged for the TEA study. A detailed description of the processes is conducted in the Supplementary Materials to precisely quantify the process inventories in relation to the selected functional unit. Note that when the plant location was considered to have an influence on the LCA or TEA results, it was assumed that the production would occur in Belgium. Of course, LCA database values as well as impact calculations are subject to uncertainties, but reasonable variations of the assumptions made in the present work do not significantly impact the results and conclusions and as such are not considered.

### 3.1. LCA Description and Methodology

Life cycle assessment (LCA) is a methodology that assesses the environmental impacts of a process or product over its whole life cycle. It quantifies the environmental impacts linked to emissions or resource consumption at each step of production. Those impacts can then be sorted in impact categories and assessed. Following the ISO 14040 and 14044 norms for LCA [39,40], the first phase is to define the goal and scope of the LCA study. This also requires the definition of a functional unit for which the impacts will be assessed. As mentioned earlier, these elements will be shared with the cost study, along with the processes’ inventories. The software used for the LCA is Simapro V9.0.0.35 (Simapro, Amersfoort, The Netherlands). The database used is Ecoinvent V3.5. The impact evaluation method is ReCiPe 2016 in a hierarchist configuration.

#### 3.1.1. Functional Unit

A functional unit is the amount of product generated for which the environmental and economic impacts are evaluated. In this study, it will be the coating of a steel cylinder’s side with a 20 µm thick chromium film. The cylinder has a height of 80 cm and diameter of 40 cm, corresponding to an area to be coated of about 1 m^2^. This functional unit has been chosen because such cylinders are commonly coated with chromium film to improve their tribological and mechanical properties for use in several sectors, such as the textile industry for example [41]. In total, 20 µm of coating has been assumed to be thick enough to see properties improvements.

#### 3.1.2. Goal and Scope

In the present case, the goal of the study is to assess and compare the environmental and techno-economic impacts for the coating of a functional unit with two deposition techniques: electrodeposition and magnetron sputtering. Regarding the environmental impacts, the main categories studied are the global warming potential and the hexavalent chromium emissions. Justifications for those choices are given in the impact assessment section (Section 4). The study of costs is included in the present work to present more nuanced trade-offs between the different technologies.

Regarding the scope, this study will only focus on the deposition step itself and will not consider: (i) cylinder manufacture, (ii) cylinder transport, (iii) deposition equipment manufacture, (iv) use, maintenance and end phases of the coated cylinder’s life cycle, (v) maintenance steps for the equipment. This study will however take into account: (i) the energy used in the coating process, (ii) the materials used, from extraction to disposal when data is available, (iii) process emissions.

Because the properties of coatings deposited with electrodeposition or with magnetron sputtering are considered equivalent, the use and the end-of-life phases of the cylinder are not taken into account.

#### 3.1.3. Inventory Analysis

Material and energy flows inventories for the coating of a functional unit are described in the Supplementary Materials. Those inventories come from the extrapolation of data from industrial and research partners as well as from literature.

#### 3.1.4. Impact Assessment and Normalization

Through the emissions listed in the inventory, impact scores in 18 different midpoint categories can be calculated using the formula:

(1) $${\mathrm{IM}}_{x}=\underset{i}{\sum }X_{i}{\;\mathrm{CF}}_{\mathrm{xi}}$$

where:

IM_x_ is the impact score of the impact category x; X_i_ is the emission i and CF_xi_ is the characterization factor of the emission i in the impact category x.

These impact scores are usually hard to interpret outside a purely comparative setting. To better show the outliers in the impact categories, the normalized results will be shown. The normalization is done by correcting the impact score with a normalization factor:

(2) $${\mathrm{NI}}_{x}=\frac{{\mathrm{IM}}_{x}}{{\mathrm{NF}}_{x}}$$

where:

NI_x_ is the normalized impact score in the x category and NF_x_ is the normalization factor of the x category.

The normalization factor, in the case of ReCiPe 2016, corresponds the global average emissions of one person in 2010. Characterization factors and normalization factors can be found on the RIVM website [42].

### 3.2. Economic Assessment

As mentioned earlier, the goal and scope of the cost study are similar to the ones identified for the LCA. The economic assessment is based on the calculation of the processes OPEX and CAPEX. To determine the operational costs for the coating of a functional unit (cost of consumables, energy…), an inventory of process streams is available in Supplementary Materials. Cost estimates are based on global market prices for the reagents and Belgian prices for water and electricity. In addition, the equipment needed for electrodeposition and their costs are estimated based on plant reports [43,44], and on personal communications with industrial experts. For magnetron sputtering, costs have been extrapolated from a project partner’s equipment bills (see Supplementary Materials). In both cases only the equipment needed for the sole purpose of coating one substrate at a time using one bath/chamber is considered. The total cost C_TOT_ is given in Equation (3).

C_TOT_ = C_OP_ + C_L_ + C_EQ_ (3)

C_OP_ = C_ELEC_ + C_MAT_ (4)

C_EQ_ = C_M_ + C_A_ (5)

where,

C_OP_ is the operating cost; C_L_ is the labor cost; C_EQ_ is the equipment cost; C_ELEC_ is the cost of electricity; C_MAT_ is the cost of the materials used; C_M_ is the maintenance cost and C_A_ is the cost of annuity.

Annuity and maintenance costs can be derived based on some hypotheses. First, a yearly maintenance cost of 2% of the equipment investment is assumed [45]. Then, equipment is assumed to run continuously during two shifts per day (16 h) and 300 days a year. Lastly, magnetron sputtering equipment is assumed to last for 15 years while electrodeposition equipment is assumed to last 10 years [46]. When prices were taken from sources using a different currency, prices were adjusted for inflation and converted to euros. The following conversion rates are used: 1 Euro (EUR) = 1.12 US Dollar (USD) = 83.3 Indian Rupee (INR).

## 4. LCA Results

In this section, environmental results are presented. First, using the previously obtained inventories, environmental impacts are worked out using the ReCiPe method to have a global view on the impacts of each process. Results are normalized corresponding to a standardized way of showing LCA outcomes.

In a second step, the different impacts of chromium production for each process are studied. As the synthesis path for the chromium source is different for each process, it is relevant to focus on the way the production impacts differ. This implies studying both metallic chromium production for magnetron sputtering as well as chromium oxide production for electrodeposition.

Finally, a discussion on the two most relevant environmental impacts is considered. From the study, it appears indeed that global warming potential and human carcinogenic toxicity are the most representative indicators to highlight differences between the two processes. Global warming potential is relevant to assess the very different energy consumptions for each process, while human carcinogenic toxicity is more representative of the harmful emissions of the electrodeposition process. However, due to the way toxicities are handled in LCA, with a large uncertainty on the link between emissions and impacts [47], the focus will then be set on the airborne emissions of hexavalent chromium rather than on the human carcinogenic toxicity itself. A discussion about those emissions and their impact will take place in Section 4.4 and Section 6, respectively.

### 4.1. Normalized Impacts

**Figure 1:**
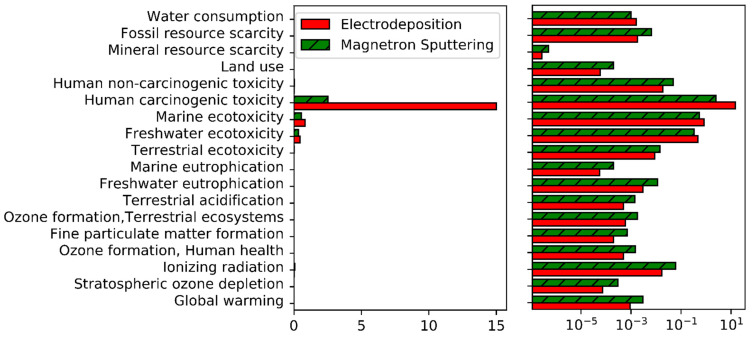
Normalized impacts of magnetron sputtering and electrodeposition for all impact categories. Left = linear scale, right = logarithmic scale.

**Figure 2:**
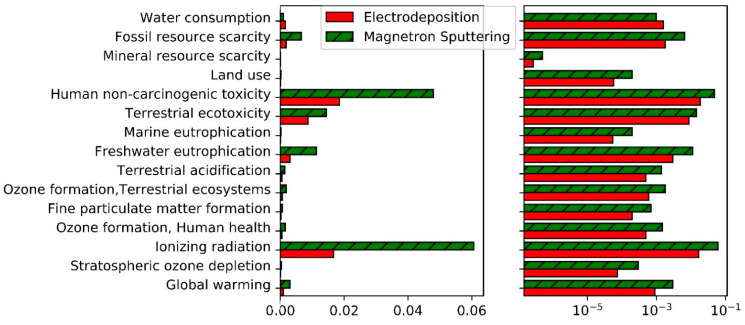
Normalized impacts of magnetron sputtering and electrodeposition for all impact categories excluding water ecotoxicities and human carcinogenic toxicity. Left = linear scale, right = logarithmic scale.

The normalized environmental impacts of each process are shown in Figure 1. Figure 2 comprises the same results excluding water ecotoxicities and human carcinogenic toxicity.

The most prominent impact category in this analysis is human carcinogenic toxicity, with an impact higher than most other impact categories by several orders of magnitude. The two other most impactful categories are freshwater and marine ecotoxicities. In the case of human carcinogenic toxicity, more than 99.9% of the impact is attributed to Cr^VI^ emissions to water during the production of the chromium source. Cr^VI^ emissions are also responsible of the majority of ecotoxicities’ impacts. As a consequence, and in agreement with the poor consideration of ecotoxicities in current LCA methodologies already mentioned earlier, the focus will be set on Cr^VI^ emissions rather than human carcinogenic toxicity in the followings.

For most other impact categories, electricity production is usually the most impactful process (note that the Belgian electricity mix has been considered in this study, which consists mainly in 45% of nuclear energy and 23% of gas power plants). Due to the high differences in power consumption between electrodeposition and magnetron sputtering, an analysis of the global warming potential of those two processes is presented in Section 4.3. Before looking in details at these two impact categories, the impact of the production of chromium will be studied in the next section.

### 4.2. Chromium Sources Comparisons

As mentioned in the Supplementary Materials, the two processes have different production paths for their source of chromium. Indeed, metallic chromium is required for magnetron sputtering while electrodeposition uses CrO_3_ salts. Different production paths then lead to different environmental impacts which have been retrieved from the Ecoinvent database and are given below. It should be noted that metallic chromium is considered to come from aluminothermy at 75% and from electrolysis at 25%. The same amount of chromium atoms is considered, leading to different weight depending on their form: 520 g of metallic Cr and 1 kg of CrO_3_.

**Figure 3:**
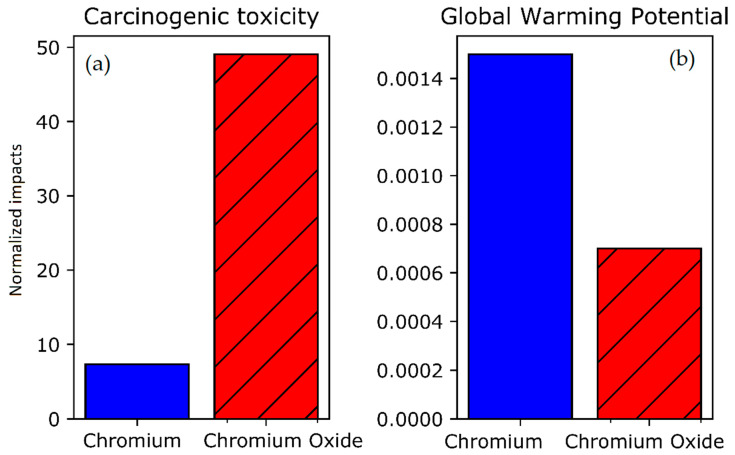
(**a**) Human carcinogenic toxicity of the production of metallic chromium vs. chromium oxide (**left**, normalized). (**b**) Global warming potential of the production of metallic chromium vs. chromium oxide (**right**, normalized).

As it can be observed in Figure 3a, due to the different reduction path for metallic chromium, the emissions of Cr^VI^, and consequently the human carcinogenic toxicity, are lower. However, Figure 3b shows that the greenhouse gases emissions are more important for metallic chromium, mainly due to the electricity, heat and aluminum needed for the reduction of the metal oxides.

In conclusion, the production of metallic chromium used in magnetron sputtering, while more energy intensive, produces less hexavalent chromium emissions than chromium oxide production, used in electrodeposition. Using this insight, a more detailed analysis of the most relevant environmental impacts can be made for the two processes in the next sections.

### 4.3. Global Warming Potential

**Figure 4:**
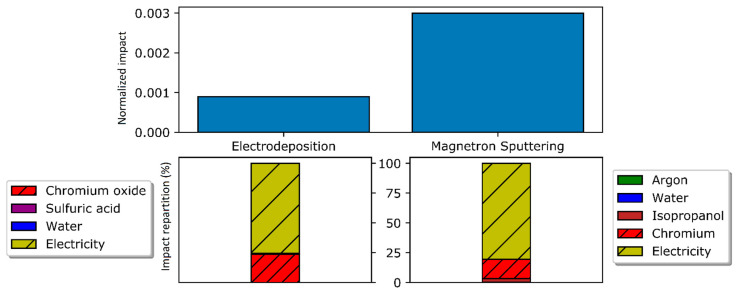
**Top**: Normalized GWP for electrodeposition and magnetron sputtering. **Bottom left**: Impact distribution for magnetron sputtering sub-processes. **Bottom right**: Impact distribution for electrodeposition sub-processes.

First, the normalized global warming potential (GWP) for both processes is given below (Figure 4, top). As it can be expected, the higher power consumption for magnetron sputtering combined to the increased greenhouse gases emissions for metallic chromium production lead to a higher global warming potential (Figure 4, bottom left), even though the chromium production itself has a relatively low impact compared to electricity production. As for the electrodeposition process, similar distributions can be observed (Figure 4, bottom right). It should also be noted that wastewater treatment is a negligible sub-process in that impact category. All in all, global warming potential is three times higher for magnetron sputtering compared to electrodeposition. It should be noted that a decarbonized energy source could mitigate this drawback largely.

### 4.4. Hexavalent Chromium Emissions

There are two stable forms of chromium in nature: Cr^III^ and Cr^VI^. While the former has low mobility and is harmless to most organisms, the latter is highly toxic over long exposure [48]. Its reduction in cells causes damage to the DNA, which leads to increased risks of cancer [49]. As mentioned earlier, Cr^VI^ emissions are the main factor in toxicity assessment. Therefore, this section will focus exclusively on the hexavalent chromium emissions. The different emission pathways for chromium have different effects on the human population and the environment. Cr^VI^ emissions can be put in two compartments: airborne and waterborne emissions. Both hexavalent chromium pathways emissions will be listed.

On one side, airborne chromium (i.e., droplets produced during industrial processes) affects the workers mainly during occupational exposure. Chronic exposition leads to an increase of respiratory cancers. On the other side, waterborne chromium (i.e., untreated plating solution, waste from chromium production, etc.) can affect organisms differently depending on the environment where it is emitted. For example, Cr^VI^ in rivers and freshwater can potentially harm the wildlife, depending on Cr^VI^ concentration [50]. Hexavalent chromium in soil can harm the organisms in the ground [51]. It may also end up in drinking water, where ingestion of hexavalent chromium could potentially lead to increased risk of cancer in the digestive system [52,53], although its effect is still debated due in part to rapid reduction of Cr^VI^ in the oral tract [54]. While Cr^VI^ is harmful to many organisms, it tends to be reduced relatively quickly in nature to Cr^III^, whose toxicity is dramatically lower, or at least not yet proven isolated from hexavalent chromium [55,56].

**Figure 5:**
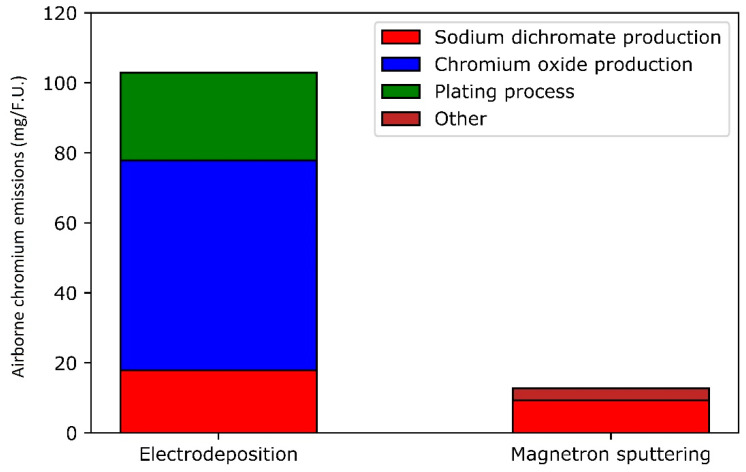
Hexavalent chromium airborne emissions.

More detailed data than the Ecoinvent database are available for Cr^VI^ production and serve to the present discussion. Indeed, a more complete inventory of chromium trioxide production has been made by Harscoet and Froelich [57], specifically for the anodizing process that requires chromic acid made from chromium trioxide. Regarding the metallic chromium production, emissions are retrieved from the Ecoinvent database. The airborne emissions are shown in Figure 5. The total emissions of airborne hexavalent chromium amount to 102.95 mg per functional unit for electrodeposition and to 12.7 mg for magnetron sputtering. Those emissions are noticeably lower for magnetron sputtering because of the synthesis path for metallic chromium and the emission-free nature of the deposition process itself. In comparison, the plating process accounts for around one quarter of the total emissions in the electrodeposition process. Most of the emissions come from the production of chromium trioxide.

**Figure 6:**
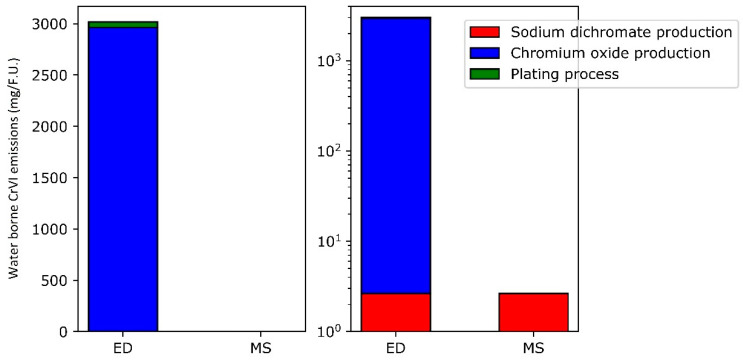
Hexavalent chromium waterborne emissions. **Left** = linear scale, **right** = logarithmic scale.

Concerning the waterborne emissions of the electrodeposition process, they only appear in two processes: direct emission during chromium oxide production and the emissions related to the treatment of wastewater containing hexavalent chromium. Harscoet and Froelich [57], based on Kowalski et al.’s work [58], predict a Cr^VI^ discharge in water in the order of 13 kg per ton of chromium trioxide produced. In the present work, that would amount to 2.96 g of emissions per functional unit produced. The rest of Cr^VI^ emissions comes from wastewater treatment. Most of that wastewater originates from the plating itself, for which the hexavalent chromium emission is assumed to be 54.5 mg (see Supplementary Materials). In the magnetron sputtering process, the only source for waterborne emissions is the production of dichromate. That process produces wastewater containing hexavalent chromium. It can be worked out that, for one functional unit, about 2.64 mg of hexavalent chromium is released in water for the production of the amount of sodium dichromate needed to produce the metallic chromium. The waterborne emissions are summarized in Figure 6.

In conclusion, the different synthesis paths for the chromium source greatly impact the emissions for each process. Mainly, the absence of chromium trioxide manufacture makes both the waterborne and airborne Cr^VI^ emissions for magnetron sputtering negligible compared to electrodeposition. It should be noted that chromium oxide, whose production leads to most of the hexavalent chromium emissions, is not manufactured in Europe anymore [59]. Consequently, most of the impact is “transferred” to other regions, with potentially less stringent regulations and Occupational Exposure Limits (OEL). This could seriously worsen the environmental impact of chromium oxides’ manufacture.

## 5. Economic Considerations

Beyond environmental considerations, economic assessment is critical when comparing technologies. The techno-economic assessment is split in two parts: the operating costs, mostly due to electricity and reagents consumption, and the capital costs. Costs for the wastewater treatment of the electrodeposition process will only include the reagents used and not the labor or equipment required.

### 5.1. Operating Costs

**Figure 7:**
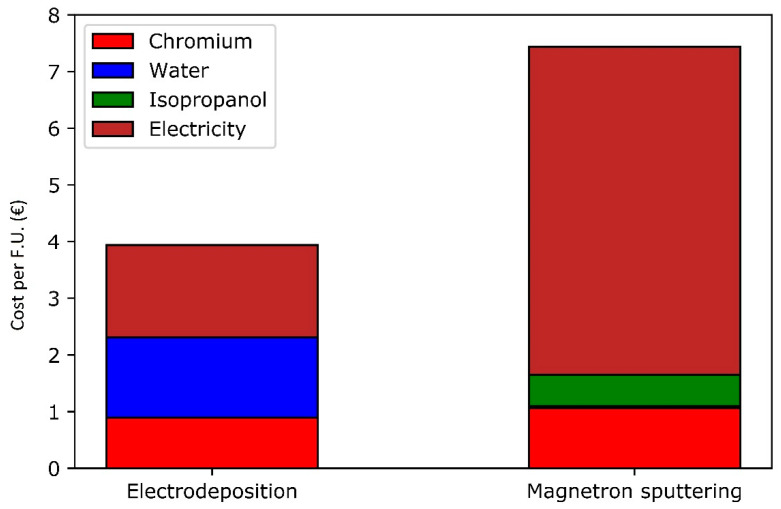
Operating costs for one functional unit for electrodeposition and magnetron sputtering (salaries excluded).

Cost of consumables for one functional unit have been estimated from catalogues and European market reports. Electricity cost has been derived from Belgian electricity costs for an industrial application. The distribution of operating costs is shown in Figure 7. Negligible costs (EUR < 0.05 per functional unit) have been omitted. Due to the high uncertainties, labor costs will be considered separately.

With a greatly increased electricity consumption for magnetron sputtering compared to electrodeposition, the costs are also higher as electricity is one of the most important drivers to the operating cost in both processes. Chromium raw materials are the second main contributor to operating cost (salaries excluded) for magnetron sputtering, while water takes the second rank in the electrodeposition process.

With a consumable cost of EUR 7.5 per functional unit for magnetron sputtering compared to electrodeposition’s EUR 4, the most economic option tends to be the latter, due to the electricity consumption. However, labor costs should also be considered as they are usually important costs in Western Europe, accounting for about 75% of operating costs for an electrodeposition process in Belgium [46]. For both coating processes, it is assumed that one worker is enough to operate the coating process for one functional unit. However, the work time needed for each process differs. The work time of an operator can be split into two main parts: (i) preparation of the substrate and rectification (which is a post-treatment step) and (ii) monitoring of the coating process.

**Figure 8:**
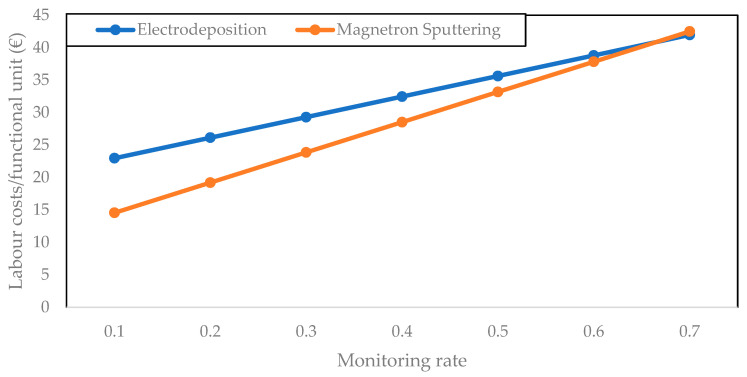
Sensitivity analysis: labor costs increase with monitoring rate.

For both processes the amount of work needed for those two operations will vary. In the case of electrodeposition, due to the higher number of preparation steps and to the rectification required, the coating process takes more work time. This duration is here assumed to be 30 min for the electrodeposition process, while it is estimated at 15 min for magnetron sputtering. Regarding the time spent on process monitoring, the worker needs to be present only during a fraction of the coating step. Due to the higher deposition rate of electrodeposition, this duration is thus lower for a given monitoring rate (fraction of coating operation time for which operator attention is required). However, the impact of this lower duration on the total labor cost varies with the assumption that is made on the monitoring rate. A sensitivity analysis of the labor cost in function of the monitoring rate is then included in Figure 8, evidencing that the labor costs increase faster with the assumed monitoring rate for the magnetron sputtering process due to its longer duration. Gross hourly salary is assumed to be EUR 39.6 [60].

When comparing Figure 7 and Figure 8, it clearly appears that total operating costs are mainly due to labor costs, even if a low monitoring rate is assumed. The dominating nature of labor costs makes the higher consumables cost of magnetron sputtering almost irrelevant. If a low monitoring rate is assumed for the coating step, magnetron sputtering could be considered more interesting due to the lower total duration of preparation steps. This changes if a higher monitoring rate is assumed, due to the longer deposition times of magnetron sputtering.

### 5.2. Equipment Costs

**Figure 9:**
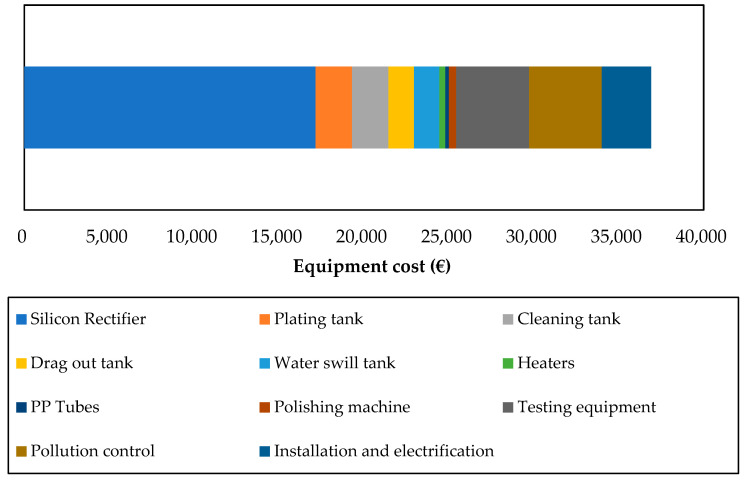
Cost breakdown of electrodeposition equipment (€).

Besides operating costs, equipment costs play an important role in the economic feasibility of a process. In the present study and for the considered functional unit, it has been assumed that working capital and plant infrastructure are not taken into account. Indeed, working capital does not affect the economic viability of a project as it does not depreciate, while plant infrastructure is neglected because its impact is supposed to be similar for both processes. The cost breakdown for electrodeposition is given in Figure 9, mainly based on MSME-DI [44], and some estimations based on communications with industrial experts and equipment catalogs.

It should be noted that there is a fairly important variation of prices between equipment from different sellers (e.g., current rectifiers from Ato vs. XingTongLi). However, the fact that the current rectifier (needed to convert a large amount of alternative current to direct current) is one of the biggest contributors, as well as its indicated price range, is confirmed by a first-hand source [46].

**Figure 10:**
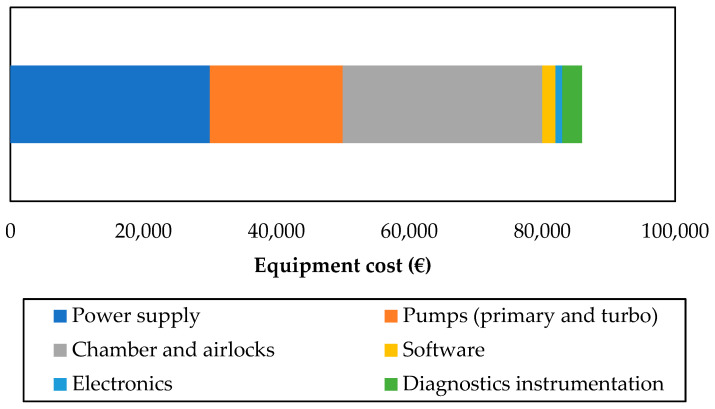
Magnetron sputtering equipment cost breakdown (€).

A cost breakdown for magnetron sputtering is given in Figure 10. The equipment cost has been estimated from a project partner’s equipment bills [61]. Power supply unit, pumps and the vacuum chamber all have approximately the same impact on the equipment cost.

It should be noted that, while this study estimates the total cost at around EUR 85,000, installations of similar sizes and functions have been found in catalogues with higher prices (EUR 120,000 to 210,000 or higher for specific applications [62]. As a consequence, the magnetron sputtering CAPEX given in this study might be underestimated, especially if specific design requirements are considered.

**Figure 11:**
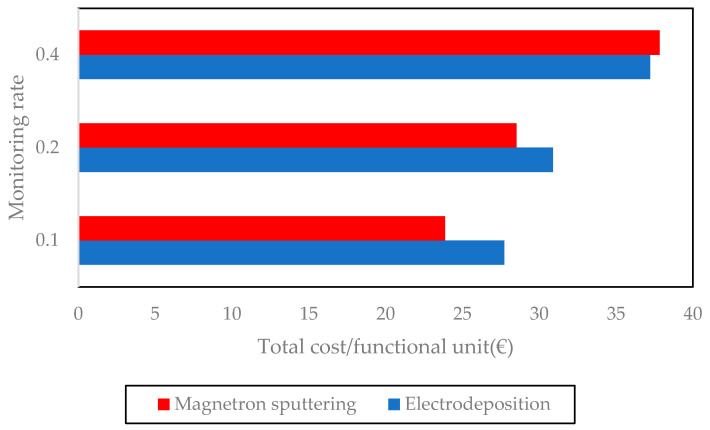
Total cost per functional unit with varying monitoring rate.

Following the assumptions made in Section 3.2, a yearly production of 6000 functional units for electrodeposition and 4114 for magnetron sputtering are obtained. This leads to a total annuity and maintenance costs of EUR 0.75 per functional unit for electrodeposition and of EUR 1.81 for magnetron sputtering. This is in line with the larger capital expenses and the lower deposition rate of magnetron sputtering identified earlier. Total costs per functional unit are given in Figure 11 for different monitoring rate assumptions.

While total cost can be lower for magnetron sputtering at low monitoring rates, it also appears from the techno-economic assessment that the operating expenses are the major cost factor, and that the labor cost plays the biggest part in these costs. Given the uncertainty on estimated operating times, more research would be needed to validate these results. Nevertheless, it clearly appears that the equipment costs have a very small impact on the total coating cost per functional unit, and that the cost per functional unit depends more on the monitoring rate rather than on the selected deposition technology.

## 6. Discussion

In this section, the environmental results of the comparison between magnetron sputtering and electrodeposition are presented first with a local approach, and then their wider implications are discussed. Following that discussion, the economic results are considered.

### 6.1. Environmental Results and Fate of Hexavalent Chromium

Firstly, the environmental conclusions obtained in this present paper are in line with similar literature [38]: more than 3 times as much energy is required for the same thickness obtained by magnetron sputtering (3.7 times as much energy needed for MS in Benveniste’s work compared to 3.6 times in the present work), and less overall emissions. The environmental and health impacts associated with each technology presently studied mainly differ in two ways: energy consumption and chromium emissions. Overall, due to the higher energy consumption, the impact is higher in most categories (including global warming potential) for magnetron sputtering. However, the greater hexavalent chromium emissions over the life cycle of the chromium trioxide make electrodeposition worse in the categories having the most prominent normalized impact (ecotoxicities and human carcinogenic toxicity). However, the use of a standard impact method for the evaluation of hexavalent chromium toxicity is not enough for a proper consideration of this impact because of the high uncertainties associated to the ecotoxicity assessment, especially for metals [47]. Additionally, discrepancies between impact assessment methods are present. From this point of view, it can be argued that, for a given metal, it is more accurate and insightful to discuss its different emission pathways and their local effect on the environment, human population or wildlife, rather than applying a generic impact evaluation method. For instance, Hedberg et al. have shown us how to make a more nuanced approach to hexavalent chromium impact assessment [63]. By considering different scenarios of Cr^VI^ reduction in the environment, they show how the impact of Cr^VI^ emissions can vary locally.

Following that statement, the first point of discussion is the fate of the contaminant in the local environment, in this case, hexavalent chromium. Hexavalent chromium, while highly toxic, tends to quickly be reduced to its trivalent state in a “typical” environment. Indeed, the presence of ferrous ions (present in a number of naturally occurring minerals), hydroxyl radicals, sulfites or even microbes or humic acid are all factors that increase the rate of reduction of hexavalent chromium [64,65]. That rate of reduction is such that the timescale for the effective “neutralization” of hexavalent chromium in those cases is extremely short compared to typical environmental hazards: depending on species present, it could go from a few hours to a month in typical conditions. As such, the nature of the environment greatly influences the impact of hexavalent chromium. It should be noted that some studies (e.g., [66]) show soil chromium contamination decades after industrial exploitation due to specific soil conditions, even if the impact is contained to a certain area thanks to the reduction mechanisms described above, which limit the migration of unreduced Cr^VI^ species. All in all, the effect tends to be relatively localized, both in terms of spatial and temporal resolution, which should then be taken into account with a characterization factor suited for each local application.

### 6.2. Environmental and Health Effects

Even if an effect is localized, it can, nevertheless, be extremely harmful. This leads into the second point of discussion which is the effect of the contaminant on humans or wildlife. As mentioned earlier in this paper, it mainly affects humans through airborne droplets, although contamination through drinking water or consumption of contaminated fish is possible. Several studies ([48,49,67]) have shown the impact of airborne emissions on chromium plant workers. The main way that hexavalent chromium can negatively impact human lives is indeed the increased risk of cancer, which fluctuates depending on exposure and Cr^VI^ concentration. The main way to assess this increased risk is to find out the number of workers potentially affected with a form of respiratory cancer due to their occupation. To that end, 3 figures must be worked out: the number of workers in the chromium industry, the number of workers that statistically should be affected by cancer independently of their occupation and the increase of cases directly caused by their occupation (with a statistically significant number of observed cases compared to the number of expected cases). For the cohort of workers studied in Gibb’s work [48], the number of observed respiratory cancer cases over the number of expected cases was of 1.64.

Transposing this calculation to a higher level, an estimated 32,000 workers are directly involved with working with chromium trioxide in Europe [68]. In order to have a first figure for comparison, the lifetime risk of contracting lung cancer was reported for a region of Switzerland to be of 8% for a man and of 4% for a woman [69]. It is in accordance with other studies and confirms that Swiss cancer rates are close to the European standard [70]. Then, it should be noted that most workers in the field are male [48]. Assuming this basic level of risk independently of occupation, the workers working in chromium industries would be expected to see 2560 cases of lung cancer. However, if an increase of observed cases of 64% is assumed like in Gibb’s work [48], this number of cases would go up to 4198 cases. With those assumptions, the use of CrO_3_ would be directly responsible of around 1638 lung cancers (2% of workers) in Europe. One should note that the assumptions made above are over-generalizing and should not considered as highly accurate due to the variability between data origin, regulations, etc. However, this figure gives a rough estimate of the potential risks for workers and related impact on public health organizations. Additionally, as mentioned in an earlier section, a large amount of Cr^VI^ emissions come from the production of chromium trioxide rather than from the plating process itself, so that the impact would be even greater for workers in the countries in which that chemical is produced. Following Figure 5 and Figure 6, the lower rate of emissions in the magnetron sputtering life cycle would most probably decrease such health risks.

### 6.3. Cost and Technical Considerations

Concerning cost estimations, while it is undeniable that magnetron sputtering equipment has a higher equipment cost as well as a higher energy cost, the lower time of pre- and post-treatments compared to electrodeposition is an indication for potential lower labor costs for magnetron sputtering. Further analysis in plant operating conditions is required to draw that conclusion. In addition, magnetron sputtering being a less industrially developed technology as well as better suited to automation [30], it could benefit from a higher learning rate leading to lower its costs compared to electrodeposition. Moreover, depending on the electrodeposition plant’s installations, subcontracting of the wastewater treatment could be a potentially expensive additional cost. With those considerations, magnetron sputtering could be less expensive than electrodeposition in the foreseeable future.

Finally, several technical advantages of each technology have been omitted to streamline the assessment process. For electrodeposition, for example, the ability to use the technology to remove former coatings and make repairs on work pieces has not been considered. That application actually makes up more than half of the work flow of plating industries. Then, regarding magnetron sputtering, the technology’s polyvalence is not taken into account. Indeed, besides pure metallic chromium coatings deposited by magnetron sputtering, other high-performance coatings can only be deposited by magnetron sputtering, possibly making this technology even more competitive when compared to electrodeposition. For example, carbides or nitrides deposited by magnetron sputtering have a hardness greater than chromium coatings [71], meaning that the same amount of protection can be obtained with thinner coatings. As an illustration, CrN has a Vickers hardness twice as important as plated chromium for example [72,73].

## 7. Conclusions

The present work has performed an environmental and cost assessment for the case of hard chromium coatings, leading to the following conclusions: while magnetron sputtering can greatly decrease the hexavalent chromium emissions over the whole process, its larger electricity consumption increases the emissions of greenhouse gases during the process due to the selected electricity mix (Belgian mix) that entails a large use of fossil fuels. Additionally, although the magnetron sputtering process itself is costlier in terms of consumables and equipment, it can be economically competitive despite its lower deposition rate thanks to lower labor costs. Moreover, as magnetron sputtering further evolves, its drawbacks could be mitigated: recent developments have shown that better deposition rates are possible with segmented targets or heated targets/substrates for example. Furthermore, if the energy mix tends to be decarbonized, most of the environmental drawbacks of magnetron sputtering will be greatly mitigated, making it even more appealing compared to electrodeposition. As a consequence, it clearly appears that magnetron sputtering suffers from operational drawbacks (mostly due to lower deposition rate) compared to the benchmark electrodeposition, but nevertheless appears as a promising technology from combined environmental and economic perspectives once the right developments have been made.

This paper opens a certain number of perspectives. For example, the study of high-performance coatings and further magnetron sputtering improvements such as High-Power Impulse Magnetron Sputtering (HiPIMS) [74], could be an additional development of the present work. In such case, a change in the functional unit from a certain amount of coating to a technical functionality (e.g., improvement in tool life time) could more fairly assess technologies and would most certainly benefit to magnetron sputtering, for which a larger range of coatings may be available. Finally, if the goal is to find alternatives to electrodeposition, it should be noted that other coating technologies, such as HVOF [18,75], also look promising based on the cost analysis and coatings performance, so they would deserve to be analyzed under the same conditions. If anything, this study emphasizes the fact that a nuanced framework is indeed needed to compare technologies under a fair lens, and that parallel cost and life cycle assessment provide a strong basis for evaluating competing technologies.

Furthermore, the present study has confirmed that insightful information can be gained both from a study of environmental impacts as well as from a study of process economics and that a fair comparison of technologies should consider both aspects. Life cycle and techno-economical assessments are clearly not redundant as they highlight different but equally important aspects of competing technologies. Performing them in parallel makes the evaluation more relevant but also more efficient, as the same inventory can be used for both assessments and trade-offs between cost and environmental impacts may be identified in an easier way.

## Data Availability

Data supporting reported results can be found in the Supplementary Materials document: process description and life cycle inventory.

## Supplementary Materials

The following Supplementary Materials are available online at https://www.mdpi.com/article/10.3390/ma14143823/s1, Supplementary Materials document: process description and life cycle inventory.

## References

[1] Statista (2021). Smartphone Users Worldwide 2016–2021. www.statista.com/statistics/330695/number-of-smartphone-users-worldwide/.

[2] Dudley B. (2020). BP Statistical Review of World Energy.

[3] Bodaghi H., Mostofi Y., Oromiehie A., Zamani Z., Ghanbarzadeh B., Costa C., Conte A., Del Nobile M.A. (2013). Evaluation of the photocatalytic antimicrobial effects of a TiO_2_ nanocomposite food packaging film by in vitro and in vivo tests. LWT Food Sci. Technol..

[4] Kaviyarasu K., Mariappan A., Neyvasagam K., Ayeshamariam A., Pandi P., Palanichamy R.R., Gopinathan C., Mola G.T., Maaza M. (2017). Photocatalytic performance and antimicrobial activities of HAp-TiO_2_ nanocomposite thin films by sol-gel method. Surf. Interfaces.

[5] Khamseh S., Alibakhshi E., Mahdavian M., Saeb M.R., Vahabi H., Kokanyan N., Laheurte P. (2018). Magnetron-sputtered copper/diamond-like carbon composite thin films with super anti-corrosion properties. Surf. Coat. Technol..

[6] IBIS World (2021). Metal Plating & Treating in the US—Market Size 2005–2027. https://www.ibisworld.com/industry-statistics/market-size/metal-plating-treating-united-states/.

[7] Grand View Research (2017). Automotive Chromium Market Analysis, by Application (Decorative Plating, Functional Plating), by End-Use (Two Wheelers, Passenger Vehicles, Commercial Vehicles), by Region, And Segment Forecasts, 2018–2025.

[8] Gaille B. (2018). 23 Chrome Plating Industry Statistics, Trends & Analysis. https://brandongaille.com/23-chrome-plating-industry-statistics-trends-analysis/.

[9] Raub C., Niece L., Craddock P. (1993). 23—The history of electroplating. Metal Plating and Patination.

[10] Bolelli G., Cannillo V., Lusvarghi L., Riccò S. (2006). Mechanical and tribological properties of electrolytic hard chrome and HVOF-sprayed coatings. Surf. Coat. Technol..

[11] European Commission (2020). Study on the EUs List of Critical Raw Materials, Factsheets on Non-Critical Materials.

[12] ECHA (2016). Annex XVII to REACH—Entry 47.

[13] Scientific Committee on Occupational Exposure Limits (2017). SCOEL/REC/386 Chromium VI compounds Recommendation from the Scientific Committee on Occupational Exposure Limits.

[14] Hansal W.E.G., Roy S. (2012). Pulse Plating.

[15] Farr J.P.G., Larson C. (2013). Current research and potential applications for pulsed current electrodeposition—A review. Trans. IMF.

[16] Drela I., Szynkarczuk J., Kubicki J. (1989). Electrodeposition of chromium from Cr (III) electrolytes in the presence of chromic acid. J. Appl. Electrochem..

[17] Protsenko V.S., Danilov F.I. (2014). Chromium electroplating from trivalent chromium baths as an environmentally friendly alternative to hazardous hexavalent chromium baths: Comparative study on advantages and disadvantages. Clean Technol. Environ. Policy.

[18] Legg K., Graham M., Chang P., Rastagar F., Gonzales A., Sartwell B. (1996). The replacement of electroplating. Surf. Coat. Technol..

[19] Cromomed (2015). Analysis of Alternatives: Functional Chrome Plating. https://echa.europa.eu/documents/10162/ece8b65e-aec0-4da8-bf68-4962158a4952.

[20] Grove W.R. (1852). On the electro-chemical polarity of gases. Philos. Trans. R. Soc..

[21] Greene J.E. (2017). Review article: Tracing the recorded history of thin-film sputter deposition: From the 1800s to 2017. J. Vac. Sci. Technol. A.

[22] Hull A. (1921). The magnetron. J. Am. Inst. Electr. Eng..

[23] Epke S.D., Jimenez F.J., Field D.J., Davis M.J., Dew S.K. (2009). Effect of magnetic field strength on deposition rate and energy flux in a dc magnetron sputtering system. J. Vac. Sci. Technol. A.

[24] Aubert A., Gillet R., Gaucher A., Terrat J.P. (1983). Hard chrome coatings deposited by physical vapour deposition. Thin Solid Film..

[25] Paturaud C., Farges G., Sainte Catherine M.C., Machet J. (1999). Correlation between hardness and embedded argon content of magnetron sputtered chromium films. Thin Solid Film..

[26] Rigi V.J.C., Jayaraj M.K., Saji K.J. (2020). Envisaging radio frequency magnetron sputtering as an efficient method for large scale deposition of homogeneous two dimensional MoS2. Appl. Surf. Sci..

[27] Weirather T., Czettl C., Polcik P., Kathrein M., Mitterer C. (2013). Industrial-scale sputter deposition of Cr1−xAlxN coatings with 0.21 ≤ x ≤ 0.74 from segmented targets. Surf. Coat. Technol..

[28] Sidelev D.V., Bleykher G.A., Bestetti M., Krivobokov V.P., Vicenzo A., Franz S., Brunella M.F. (2017). A comparative study on the properties of chromium coatings deposited by magnetron sputtering with hot and cooled target. Vacuum.

[29] Correia F.C., Bundaleski N., Teodoro O.M.N.D., Correia M.R., Rebouta L., Mendes A., Tavares C.J. (2018). XPS analysis of ZnO: Ga films deposited by magnetron sputtering: Substrate bias effect. Appl. Surf. Sci..

[30] Domanowski P., Wawrzak A. (2012). Automation of thin film deposition process based on magnetron sputtering. J. Mach. Eng..

[31] Sidorova S., Koupstov A.D., Pronin M.A., Radionov A., Karandaev A. (2020). Problems and solutions of automation of magnetron sputtering process in vacuum. Advances in Automation. RusAutoCon 2019, Proceedings of the Lecture Notes in Electrical Engineering 2020, Sochi, Russia, 8–14 September 2019.

[32] Kapopara J.M., Patel N.P., Kotadiya D.J., Patel A.R., Chauhan K.V., Rawal S.K. (2017). FEA Analysis of Zirconium Nitride Coatings Prepared by RF Magnetron Sputtering: CFD Approach. Mater. Today Proc..

[33] Mahieu S., Buyle G., Depla D., Heirwegh S., Ghekiere P., De Gryse R. (2006). Monte Carlo simulation of the transport of atoms on DC magnetron sputtering. Nucl. Instrum. Methods Phys. Res. Sect. B Beam Interact. Mater. Atoms.

[34] Alami J., Bolz S., Sarakinos K. (2009). High power pulsed magnetron sputtering: Fundamentals and applications. J. Alloys Compd..

[35] Krishnan N., Vardelle A., Legoux J. (2008). A life cycle comparison of hard chrome and thermal sprayed coatings: A case example of aircraft landing gears. International Thermal Spray, ITSC 2008, Thermal Spray Crossing Borders.

[36] Serres N., Hlawka F., Costil S., Langlade C., Machi F. (2011). Corrosion properties of in situ laser remelted NiCrBSi coatings comparison with hard chromium coatings. J. Mater. Process. Technol..

[37] Rodriguez R., Espada J.J., Gallardo M., Molina R., López-Muñoz M.J. (2018). Life cycle assessment and techno-economic evaluation of alternatives for the treatment of wastewater in a chrome-plating industry. J. Clean. Prod..

[38] Benveniste G., Baldo G., Perucca M., Ruggeri B. LCA comparative analysis of different technologies for surface functionalization. Proceedings of the 3rd International Conference on Life Cycle Management.

[39] European Committee for Standardisation (2006). ISO 14040—Environmental Management—Life Cycle Assessment—Principle and Framework.

[40] European Committee for Standardisation (2006). ISO 14044—Environmental Management—Life Cycle Assessment—Requirements and Guidelines.

[41] Advance Rubtech (2020). List of Hard Chrome Plated Rolls. https://rubberrollermanufacturers.com/hard-chrome-plated-roll/.

[42] National Institute for Public Health and the Environment (2020). ReCiPe Characterisation Factors and Normalization Scores. https://www.rivm.nl/en/life-cycle-assessment-lca/downloads.

[43] Small Industries Service Institute (2003). Hard Chrome Plating.

[44] MSME-DI (2011). Hard Chromium Plating Plant Report, Metal Finishing Division.

[45] Lauer M. (2008). Methodology Guideline on Techno-Economic Assessment (TEA). Report Generated in the Framework of ThermalNet WP3B Economics.

[46] De Visscher J.-L. (2019). Informal Interview about Chromium Plating in the Company le Chromage Dur.

[47] Pizzol M., Christensen P., Schmidt J., Thomsen M. (2011). Eco-toxicological impact of “metals” on the aquatic and terrestrial ecosystem: A comparison between eight different methodologies for Life Cycle Impact Assessment (LCIA). J. Clean. Prod..

[48] Gibb H.J., Lees P.S.J., Wang J., OLeary K.G. (2015). Extended followup of a cohort of chromium production workers. Am. J. Ind. Med..

[49] Park R.M., Bena J.F., Stayner L.T., Smith R.J., Gibb H.J., Lees P.S.J. (2004). Hexavalent chromium and lung cancer in the chromate industry: A quantitative risk assessment. Risk Anal..

[50] Venkatramreddy V., Vutukuru S.S., Tchounwou P.B. (2009). Ecotoxicology of Hexavalent Chromium in Freshwater Fish: A Critical Review. Rev. Environ. Health.

[51] Shahid M., Shamshad S., Rafiq M., Khalid S., Bibi I., Niazi N.K., Dumat C., Rashid M.I. (2017). Chromium speciation, bioavailability, uptake, toxicity and detoxification in soil-plant system: A review. Chemosphere.

[52] Stout M.D., Herbert R.A., Kissling G.E., Collins B.J., Travlos G.S., Witt K.L., Melnick R.L., Abdo K.M., Malarkey D.E., Hooth M.J. (2009). Hexavalent chromium is carcinogenic to F344/N rats and B6C3F1 mice after chronic oral exposure. Environ. Health Perspect..

[53] Linos A., Petralias A., Christophi C.A., Christoforidou E., Kouroutou P., Stoltidis M., Veloudaki A., Tzala E., Makris K.C., Karagas M.R. (2011). Oral ingestion of hexavalent chromium through drinking water and cancer mortality in an industrial area of Greece—An ecological study. Environ. Health.

[54] Proctor D.M., Otani J.M., Finley B.L., Paustenbach D.J., Bland J.A., Speizer N., Sargent E.V. (2011). Is Hexavalent chromium carcinogenic via ingestion? A weight-of-evidence review. J. Toxicol. Environ. Health.

[55] US EPA (1998). Toxicological Review of Trivalent Chromium.

[56] Wilbur S., Abadin H., Fay M., Yu D., Tencza B., Ingerman L., Klotzbach J., James S. (2012). Toxicological Profile for Chromium.

[57] Harscoet E., Froelich D. (2007). Use of LCA to evaluate the environmental benefits of substituting chromic acid anodizing (CAA). J. Clean. Prod..

[58] Kowalski Z., Kulczycka J., Wzorek Z. (2007). Life cycle assessment of different variants of sodium chromate production in Poland. J. Clean. Prod..

[59] European Commission (2016). Impact Assessment Accompanying the Document “Proposal for a Directive of the European Parliament and of the Council Amending Directive 2004/37/EC on the Protection of Workers from the Risks Related to Exposure to Carcinogens or Mutagens at Work(…).

[60] Eurostat (2018). Labour Costs in the EU: Hourly Labour Costs Ranged from €4.9 to €42.5 across the EU Member States in 2017. Lowest in Bulgaria and Romania, highest in Denmark and Belgium.

[61] CRM Group (2020). Informal Discussion with CRM Scientists Based on Equipment Invoices Collected at CRM Group.

[62] AJA International Inc. (2020). Sputtering Systems Catalog. www.ajaint.com/sputtering-systems.html.

[63] Hedberg J., Fransson K., Prideaux S., Roos S., Jönsson C., Wallinder I.O. (2019). Improving the life cycle impact assessment of metal ecotoxicity: Importance of chromium speciation, water chemistry, and metal release. Sustainability.

[64] Palmer C.D., Puls R.W. (1994). Natural Attenuation of Hexavalent Chromium in Groundwater and Soils.

[65] Lin C.-J. (2002). The chemical transformations of chromium in natural waters—A model study. Water Air Soil Pollut..

[66] Adam V., Loyaux-Lawniczak S., Quaranta G. (2012). Terrestrial and aquatic ecotoxicity assessment of Cr(VI) by the ReCiPe method calculation (LCIA): Application on an old industrial contaminated site. Environ. Sci. Pollut. Res..

[67] Gibb H.J., Lees P.S.J., Pinsky P.F., Rooney B.C. (2000). Lung cancer among workers in chromium chemical production. Am. J. Ind. Med..

[68] European Parliament (2020). Motion for a Resolution Pursuant to Rule 112(2) and (3) of the Rules of Procedure on the Draft Commission Implementing Decision Partially Granting an Authorisation under Regulation (EC) No 1907/2006 of the European Parliament and of the Council…. https://www.europarl.europa.eu/doceo/document/B-9-2020-0202_EN.html.

[69] Germann S., Konzelmann I., Chiolero A. (2017). Estimating the lifetime risk of cancer in one region of Switzerland: Arnaud Chiolero. Eur. J. Public Health.

[70] Lung Cancer Europe LuCe Report on Lung Cancer, LuCe. Public Communication 2016. https://www.lungcancereurope.eu/wp-content/uploads/2017/10/LuCE-Report-final.pdf.

[71] Seok J.-W., Lin R.Y., Jaheed N.M. (2001). Sputter-Deposited Nanocrystalline Cr and CrN Coatings on Steels. Surf. Coat. Technol..

[72] Hones P., Sanjines R., Levy F. (1997). Characterization of sputter-deposited chromium nitride thin films for hard coatings. Surf. Coat. Technol..

[73] Lausmann G. (1996). Electrolytically deposited hard chrome. Curr. Ind. Pract..

[74] Sarakinos K., Alami J., Konstantinidis S. (2010). High power pulsed magnetron sputtering: A review on scientific and engineering state of the art. Surf. Coat. Technol..

[75] Picas J., Forn A., Matthäus G. (2006). HVOF Coatings as an alternative to hard chrome for pistons and valve. Wear.

