# Supplementary material for: Magnetron Sputtering vs. Electrodeposition for Hard Chrome Coatings: A Comparison of Environmental and Economic Performances
Source: Materials (Basel). 2021 Jul 8;14(14):3823. doi: 10.3390/ma14143823 (PMC8307072; doi:10.3390/ma14143823)
Supplement: Supplementary file 1 [file materials-14-03823-s001.zip › materials-1255222-supplementary.pdf]

materials

MDPI

Supplementary Materials

Process Description and Life Cycle Inventory

Antoine Merlo * and Grégoire Léonard

Department of Chemical Engineering, University of Liège, Quartier Agora B6a Sart-Tilman, 4000 Liège, Belgium; g.leonard@uliege.be
* Correspondence: antoine.merlo@uliege.be

In the present section, the magnetron sputtering and the electrodeposition processes are described with more details. Assumptions will be made on the two processes in order to establish a process inventory. As a result, an inventory of process mass and energy flows is provided. This inventory will be used for both the environmental and economic impact assessment. Finally, this section will also include a discussion on the source of chromium used for the coating as it differs between the processes. Indeed, magnetron sputtering requires metallic chromium while the raw material used for electrodeposition is chromium trioxide.

I.1 Magnetron Sputtering Model

In order to establish the process inventory, it is needed to first estimate the size of the coating equipment. Based on typical magnetron sputtering chamber configuration (CRM group, 2020), the dimensions described in Table S1 are assumed. Besides the targets, the chamber is assumed to also contain the following equipment:

Motors, to rotate the substrate during operation to have a homogeneous coating thickness

Diagnostic tools, such as pressure sensors, to monitor operation.

Table S1. Chamber dimensions.

Chamber dimensions		
**Length**	0.8	m
Width	0.8	m
Height	1.2	m
Volume	0.768	m^3

A typical configuration involving 4 targets placed around the substrate, as shown in Figure S1, is assumed. A ratio "target area/coating area" of about 80% has been considered, based on typical values seen in commercial coating systems, for a total target area of 80 dm^2.

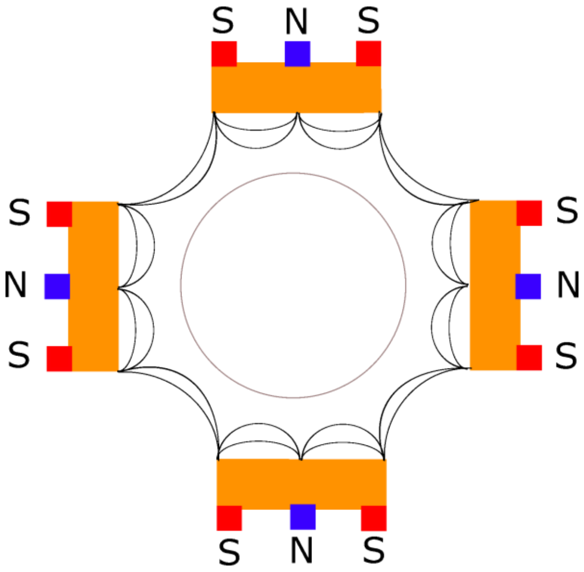

Figure S1. Chamber configuration.

The first metrics that can be worked out to build the process inventory are related to the energy consumption. There are three electricity-consuming systems in magnetron sputtering: plasma generation, vacuum pumping, and process control. Power density on the targets as well as deposition rates are assumed to be of 6.875 W/cm² and 17 μm/h respectively (Sidelev et al., 2017). The total power needed for plasma generation is then of 55 kW, and the deposition time reaches about 70 min. This deposition time will govern energy consumption. The target is assumed to not be heated.

To assess the total energy consumed during operation, the auxiliary equipment power needs will be estimated as well. Based on catalogues of pumping equipment, the pumping systems for this model are assumed to be one mechanical pump (rotary vane, e.g.) and one turbomolecular pump. The point of the mechanical pump is to obtain an intermediate vacuum for the turbomolecular pump to work in optimal gas load conditions (Mattox, 2010). The pumps lead to a total power usage of 4 kW and keep the chamber at a pressure of 0.2 Pa. Additionally, the total power for miscellaneous control equipment, cooling systems and motors has been derived from an already existing plant and estimated to 2 kW. The total power during deposition is then estimated to be 61 kW. The total electricity requirement for the coating of one functional unit would then be around 72 kWh.

Regarding the material consumption of the process, following assumptions are considered: 8 kg of water and 200 g of isopropanol are used to clean the cylinder to be coated before deposition. Moreover, during the coating process, a flow of argon of 200 standard cubic centimetres per minute flows through the chamber. This adds up to 25.12 g of argon per functional unit. Regarding the chromium consumption, the coating density has been assumed to be equal to bulk chromium density (7.19 g/cm³). This assumption can be made thanks to the higher density of magnetron sputtering coatings due to the smaller grain size (Chen, 2020). For example, Cosset et al. (1996) reported grain size for chromium deposited by magnetron sputtering between 25–50 nm while Barbato et al. (2008) observed grain sizes of at least 32.4 μm for electrodeposited chromium. We can then assume a mass for the chromium coating of 144 g.

Additionally, to take into account losses on the chamber walls, 25.5 g of chromium (15% losses) have been assumed to end up neither on the piece nor on the other targets. This chromium is then sanded off and disposed of. The final mass and energy inventory per functional unit is shown in Table S2.

Table S2. Magnetron sputtering inventory per functional unit (1 m^2 coating with 20 µm film thickness).

Flow	Amount	Unit
**Inputs**		
Argon	25.12	g
Tap water	8	kg
Isopropanol	0.2	kg
Chromium	0.1685	kg
Electricity mix, BE	71.76	kWh
**Outputs**		
Argon (to air)	25.12	g
Chromium (to soil)	25.5	g
Water to sewer	8	kg

I.2 Electrodeposition Model

As for magnetron sputtering, a single deposition bath is considered for the coating of the functional unit. Its dimensions are the same as for the deposition chamber considered for magnetron sputtering (Table S3).

Table S3. Bath dimensions.

Bath Dimensions		
Length	0.8	m
Width	0.8	m
Height	1.2	m
Volume	0.768	m^3
Top side area	0.64	m^2

The cylinder is first degreased and rinsed, then undergoes an etching (sometimes called pickling) step, where oxides are removed from its surface by immersing the piece in a chromic acid solution (Cromomed, 2015). The piece is then rinsed and goes through the plating step. During plating, venting hoods over the baths remove most of the chromium mist. After the piece is coated, it is once again rinsed before being machined (rectified) so the film has the right dimensions and is homogenous. The piece is finally rinsed one last time to remove lubricating oil and chromium flakes from its surface before being packaged and shipped to customers.

Two process steps use a non-negligible amount of power: the plating step itself due to the current that is needed to perform the redox reaction, and the heating of the bath, which must be kept at a temperature of 55 °C. Electrolytic baths are open to the ambient on their top side but are well insulated on the lateral sides, so that heat losses at the sides are neglected in first approach. The typical deposition parameters from a Belgian chromium plating company (De Visscher, 2019) are shown in Table S4.

Table S4. Deposition parameters for the electrodeposition process.

**Deposition parameters**		Unit
Bath temperature	55	°C
Deposition rate	25	μm/h
Substrate current density	40	A/dm^2
Voltage	6	V

Using those parameters for the coating process, the electricity consumption for a functional unit can be worked out, assuming electric heating like in the Belgian plant. It is described in Table S5 below. Deposition time is 48 minutes.

Table S5. Energy consumption inventory for the electrodeposition process.

**Energy Consumption**		Unit
Deposition time	0.8	h
Heat exchange coefficient (water with air flow)	50	W/m^2K
Predicted heat loss (T_ext = 20°C)	1.12	kW
Power applied to electrodes	24.13	kW
Total power usage	25.25	kW
Energy used during deposition	20.2	kWh

Regarding the materials inventory, the density of the film is assumed to be 7.020 g/cm^3 (Krishnan et al., 2008), and the coating consists in about 97.6% of bulk chromium. Deposited chromium film for the considered functional unit (cylinder with an area to be coated of 1 m^2) will then have a mass of 140 g. As CrO_3 is used as Chromium source in the electrodeposition process (see also next section), it translates then in a consumption of CrO_3 of about 292.5 g. The reduction is not complete, and its efficiency is estimated at 98% (US EPA, 1996), so 298.5 g of CrO_3 are used and 6 g end up in the spent solution. Consumption of sulfuric acid is assumed to be 1.2 g per kg of chromium (Krishnan et al., 2008; National Center for Manufacturing Science, 2000), which leads to around 0.17 g in our case study. Finally, 300 L of water are consumed during the plating process (Rodriguez et al., 2018) and 8 L per rinsing phase (Krishnan et al., 2008), leading to a total of 332 L per functional unit.

Regarding the emissions, two sources of emissions can be identified that are specific to the electrodeposition process. First, chromium is emitted during the plating process. This mainly includes the emission of plating solution droplets containing hexavalent chromium. These droplets are entrained outside of the bath with the formation of gaseous hydrogen and oxygen at the electrodes. The mist formed in the process is one of the most impactful emissions for the health of the plant workers. If inhaled, it can lead to increased risks of cancer to the respiratory system. The hexavalent chromium air emissions during the plating process have been estimated based on the US EPA (1996). Chromium emissions are estimated to 7.8 mg per ampere and per hour. In our case, that would lead to emissions of 25.1 g. Because of the use of wetting agents and venting hoods, that emission can be reduced by 99.9% to 25.1 mg. It should be noted that different studies predict different Cr^{VI} emissions. For example, emissions over a ventilated bath of 1.1 (±0.1) μg/A·h have been reported (NIOSH, 1984), which would amount to 3.54 mg over the whole plating step. For the sake of consistency between sources, the first figure will be kept and used for the process inventory.

Secondly, chromium is emitted under the form of wastewater after the plating process. This includes rinsing water as well as the spent plating solution. Cr^{VI} emissions in wastewater are adapted from the work of Rodriguez et al. (2018) and amount to 54.5 mg.

Finally, the process inventory is shown below in Table S6. The water treatment inventory of the waste produced for one functional unit is based on Rodriguez et al. (2018) and shown in Table S7.

Table S6. Plating process inputs and emissions for the coating of one functional unit by electrodeposition.

Flow	Amount	Unit	Source
**Inputs**			
Electricity	20.2	kWh	Present work
Chromium oxide	298.5	g	Present work
Sulfuric acid	0.17	g	(Krishnan et al., 2008)
Water	332	L	(Rodriguez, et al., 2018)
**Outputs**			
Lubricating oil	28	g	(Rodriguez, et al., 2018)
Airborne Cr^{VI} emissions	25.1	mg	(US EPA, 1996)
Waterborne Cr^{VI} emissions	54.5	mg	(Rodriguez, et al., 2018)

Table S7. Inputs for wastewater treatment (Rodriguez, et al., 2018)

Flow	Amount	Unit	Source
**Inputs**			
NaOH	26	g	(Rodriguez, et al., 2018)
SO_2	18	g	(Rodriguez, et al., 2018)
Sulfuric Acid	54	g	(Rodriguez, et al., 2018)
MgO	29	g	(Rodriguez, et al., 2018)
Water	19	L	(Rodriguez, et al., 2018)

I.3 Metallic Chromium and Chromium Oxide Production

In addition to having radically different processes, the chromium source differs for the two technologies: metallic chromium for magnetron sputtering and CrO_3 salts for electrodeposition. In order to complete the comparison of the coating technologies, a short description of the manufacture processes leading to the desired chromium raw material is needed.

Both metallic chromium as well as chromium oxide come from chromite ore ($FeCr_2O_4$). That chromite ore is roasted with sodium carbonate and then turned into sodium chromate via the following reactions:

$$4\,FeCr_2O_4 + O_2 \rightarrow 4\,Cr_2O_3 + 2\,FeO_3 \tag{1}$$

$$2\,Cr_2O_3 + 4\,Na_2CO_3 + 3\,O_2 \rightarrow 4\,Na_2CrO_4 + 4\,CO_2 \tag{2}$$

This compound is then leached with sulfuric acid to recover sodium dichromate ($Na_2Cr_2O_7$). Then the two processes diverge in the treatment of sodium dichromate. To produce CrO_3 salts used in electrodeposition, the dichromate is heated with sulfuric acid to 200 °C. On the other side, to obtain metallic chromium, the dichromate is heated with sulfur at around 800 °C (Aghaie-Khafri, 2014) to produce Cr_2O_3. That compound is then usually reduced to metal chromium through aluminothermy, however other metals can also be used. A recapitulative flowsheet is shown in Figure S2. The two different production paths for chromium oxide and metallic chromium have different energy requirements, emissions, and costs. This is why they need to be studied in order to have a complete comparison of magnetron sputtering and electrodeposition. In the present work, data for these two processes have been retrieved from the Ecoinvent database.

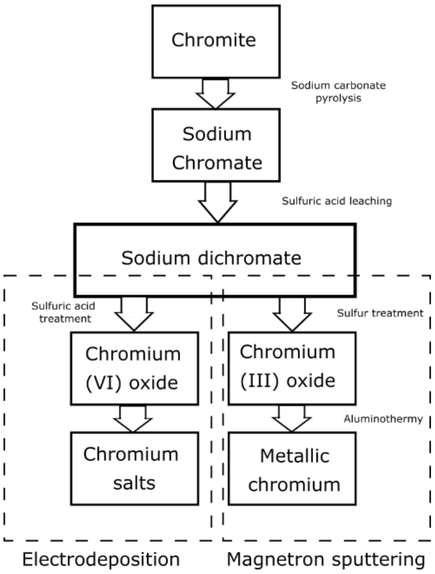

Figure S2. Treatments of chromite ore for producing the chromium raw materials used in electrodeposition and magnetron sputtering.

References

1. Aghaie-Khafri, M.; Navazany, S. Producing Cr2O3 nanooxide from chromite concentrates *Micro Nano Lett.* **2014**, *9*, 351–354.
2. Barbato, S.; Ponce, J.; Jara, M.; Cuevas, J.; Egana, R. Study of the effect of temperature on the hardness, grain size, and yield in electrodeposition of chromium on 1045 steel. *J. Chil. Chem. Soc.* **2008**, *53*, 1440–1443.
3. Chen, E. (2020). Thin film deposition course. [Online] Available online: www.mrsec.harvard.edu/education/ap298r2004/Erli%20chenFabrication%20II%20-%20Deposition-1.pdf (Accessed 12 April 2020).
4. Cosset, F.; Contoux, G.; Celerier, A.; Machet, J. Deposition of corrosion-resistant chromium and nitrogen-doped chromium coatings by cathodic magnetron sputtering. *Surf. Coat. Technol.* **1996**, *79*, 25–34.
5. CRM Group. *Informal Discussion with CRM Scientists Based on Equipment Invoices Collected at CRM Group*; 2020.
6. De Visscher, J.-L. *Informal Interview about Chromium Plating in the Company le Chromage Dur*; 2019.
7. Krishnan, N.; Vardelle, A.; Legoux, J. A life cycle comparison of hard chrome and thermal sprayed coatings a case example of aircraft landing gears. *International Thermal Spray, ITSC 2008, Thermal Spray Crossing Borders*, Maastricht, The Netherlands, 14–16 May 2007.
8. Mattox, D. Handbook of Physical Vapour Deposition (PVD) processing; Elsevier: Amsterdam, The Netherlands, 2010.
9. National Center for Manufacturing Science. *Benchmarking Metal Finishing*; National Center for Manufacturing Science: Ann Arbor MI, USA, 2000.
10. NIOSH. *Control technology assessment : Metal plating and cleaning operations,* U.S. Department of health and human services: Cincinnati, OH, USA, 1984.
11. Rodriguez, R.; Espada, J.J.; Gallardo, M.; Molina, R.; López-Muñoz, M.J.. Life cycle assessment and techno-economic evaluation of alternatives for the treatment of wastewater in a chrome-plating industry. *J. Clean. Prod.* **2018**, *172*, 2351–2362.
12. Sidelev, D.V., Bleykher, G.A., Bestetti, M., Krivobokov, V.P., Vicenzo, A., Franz, S. and Brunella, M.F. (2017). A comparative study on the properties of chromium coatings deposited by magnetron sputtering with hot and cooled target. *Vacuum,* **2017**, *143*, 479–485.
13. US EPA. *Emission Factor Documentation for AP-42, Section 12.20. Electroplating, Final Report,* US Environment Protection Agency: Research Triangle Park, NC, USA, 1996.
